# Pentoxifylline Reverses Chronic Experimental Chagasic Cardiomyopathy in Association with Repositioning of Abnormal CD8^+^ T-Cell Response

**DOI:** 10.1371/journal.pntd.0003659

**Published:** 2015-03-19

**Authors:** Isabela Resende Pereira, Glaucia Vilar-Pereira, Otacilio Cruz Moreira, Isalira Peroba Ramos, Daniel Gibaldi, Constança Britto, Milton Ozório Moraes, Joseli Lannes-Vieira

**Affiliations:** 1 Laboratório de Biologia das Interações, IOC-Fiocruz, Rio de Janeiro, Brazil; 2 Laboratório de Biologia Molecular e Doenças Endêmicas, IOC-Fiocruz, Rio de Janeiro, Brazil; 3 Laboratório de Cardiologia Celular e Molecular, Instituto de Biofísica Carlos Chagas Filho, Universidade Federal do Rio de Janeiro, Rio de Janeiro, Brazil; 4 Laboratório de Hanseníase, IOC-Fiocruz, Rio de Janeiro, Brazil; Albert Einstein College of Medicine, UNITED STATES

## Abstract

**Background:**

Chronic chagasic cardiomyopathy (CCC), the main clinical sign of Chagas disease, is associated with systemic CD8^+^ T-cell abnormalities and CD8-enriched myocarditis occurring in an inflammatory milieu. Pentoxifylline (PTX), a phosphodiesterase inhibitor, has immunoregulatory and cardioprotective properties. Here, we tested PTX effects on CD8^+^ T-cell abnormalities and cardiac alterations using a model of experimental Chagas’ heart disease.

**Methodology/Principal Findings:**

C57BL/6 mice chronically infected by the Colombian *Trypanosoma cruzi* strain and presenting signs of CCC were treated with PTX. The downmodulation of T-cell receptors on CD8^+^ cells induced by *T*. *cruzi* infection was rescued by PTX therapy. Also, PTX reduced the frequency of CD8^+^ T-cells expressing activation and migration markers in the spleen and the activation of blood vessel endothelial cells and the intensity of inflammation in the heart tissue. Although preserved interferon-gamma production systemically and in the cardiac tissue, PTX therapy reduced the number of perforin^+^ cells invading this tissue. PTX did not alter parasite load, but hampered the progression of heart injury, improving connexin 43 expression and decreasing fibronectin overdeposition. Further, PTX reversed electrical abnormalities as bradycardia and prolonged PR, QTc and QRS intervals in chronically infected mice. Moreover, PTX therapy improved heart remodeling since reduced left ventricular (LV) hypertrophy and restored the decreased LV ejection fraction.

**Conclusions/Significance:**

PTX therapy ameliorates critical aspects of CCC and repositioned CD8^+^ T-cell response towards homeostasis, reinforcing that immunological abnormalities are crucially linked, as cause or effect, to CCC. Therefore, PTX emerges as a candidate to treat the non-beneficial immune deregulation associated with chronic Chagas' heart disease and to improve prognosis.

## Author Summary

Chronic chagasic cardiomyopathy (CCC) is the main clinical manifestation of Chagas disease (CD), a neglected illness caused by the protozoan parasite *Trypanosoma cruzi*. More than hundred years after its discovery, CD continues to be a public health problem and millions of chronically infected people wait for an effective treatment. Chagasic cardiomyopathy is associated with CD8^+^ T-cell-enriched myocarditis, fibrosis and cardiac electrical and structural abnormalities, frequently progressing to heart failure. Presently, the available therapies only mitigate symptoms of CCC. Abnormalities in CD8^+^ T-cell compartment are present in CCC patients. Recently, we described the importance of CD8^+^ T-cells in the pathogenesis of CCC. Therefore, our proposal was to interfere with abnormalities of CD8^+^ T-cells glimpsing a better prognosis for CCC. Using PTX, an affordable drug with immunomodulatory properties on T-cells and cardioprotective effects in non-infections disease, we bring a therapeutic candidate for treating CCC. PTX therapy downmodulated detrimental CD8^+^ T-cells and promoted *T*. *cruzi*-specific interferon-gamma-producing T-cells. Importantly, chronic chagasic electrical and echocardiographic alterations were reversed by PTX therapy. Future studies may test the use of PTX combined with trypanocidal drug or as a vaccine adjuvant to improve the quality of life of chronic CD patients.

## Introduction

Chagas disease (CD), a neglected tropical disease caused by the protozoan parasite *Trypanosoma cruzi*, affects 6 to 8 million people in Latin America [1]. The cardiac form, the most frequent clinical manifestation of CD, is characterized by fibrosis with remodeling of the myocardium and vasculature, which commonly progresses to heart failure [2]. The chronic chagasic cardiomyopathy (CCC) is a low-grade CD8^+^-enriched myocarditis occurring in an inflammatory cytokine-embedded milieu [3–5]. Abnormal CD8^+^ T-cell function may contribute to systemic inflammatory profile and cardiac tissue lesion in the chronic phase of *T*. *cruzi* infection [6–10]. Regardless their importance for *T*. *cruzi* host resistance [11], CD8^+^ T-cells gained particular attention as the major component of myocarditis in acute [12] and chronic [9,13] experimental *T*. *cruzi* infection and in chagasic patients with CCC [3,4,14]. Recently, we proposed that interferon-gamma (IFNγ)^+^ CD8^+^cells exert a beneficial role, whereas perforin (Pfn)^+^ CD8^+^ cells take part in *T*. *cruzi*-induced heart injury [9]. Additionally, we proposed that a proper therapeutic tool could interfere with distinct CD8^+^ T-cell populations hampering heart injury [9]. Indeed, CD8^+^ T-cell abnormalities and systemic inflammatory profile were reduced by administration of the anti-tumor necrosis factor (TNF) antibody Infliximab to a model of Chagas’ heart disease [15]. These findings unveiled that reversal of systemic immunological unbalance is a rational pathway to be explored to improve the prognosis of Chagas’ heart disease.

The methylxanthine pentoxifylline (PTX) is a phosphodiesterase inhibitor commonly used to treat peripheral vascular diseases. PTX also shows therapeutic potential as an anti-inflammatory and anti-tumor agent [16]. PTX has previously been proposed as an adjuvant therapeutic tool for leishmaniasis, a protozoan disease with an extensive inflammatory component [17]. Further, in non-infectious heart disorders PTX has shown cardioprotective effects in association with reduced plasma levels of TNF [18,19].

Given the lack of an effective specific therapy, CCC is treated similarly to all other heart failure syndromes using therapies to mitigate symptoms [2]. It is proposed that CCC pathogenesis relies on a parasite-driven systemic inflammatory profile, which may reverberate in the cardiac tissue and contribute to heart dysfunction [5,10,15,20,21]. Therefore, PTX arises as a therapeutic tool to interfere with immunological unbalance and to improve the progressive functional compromise of the heart in CD. Here we tested the effects of PTX on hallmarks of immunological and heart alterations detected in CD, using a model of CCC associated with high TNF expression and CD8^+^ T-cell abnormalities [9,15,22].

## Material and Methods

### Ethical information

This study was carried out in strict accordance with the recommendations in the Guide for the Care and Use of Laboratory Animals of the Brazilian National Council of Animal Experimentation (http://www.cobea.org.br/) and the Federal Law 11.794 (October 8, 2008). The Institutional Committee for Animal Ethics of Fiocruz (CEUA-Fiocruz-L004/09; LW-10/14) approved all experimental procedures used in the present study. All presented data were obtained from three (D2–4) independent experiments (Experiment Register Books #41 and 49, LBI/IOC-Fiocruz).

### Experimental infection

Mice obtained from the animal facilities of the Oswaldo Cruz Foundation (CECAL/Fiocruz, Rio de Janeiro, Brazil) were housed under specific pathogen-free conditions in a 12-h light-dark cycle with access to food and water *ad libitum*. Five- to 7-week-old female C57BL/6 (H-2^b^) and C3H/He (H-2^k^) were intraperitoneally infected with 100 blood trypomastigotes (bt) of the Type I Colombian strain [23] of *T*. *cruzi*, and parasitemia was employed as a parameter to establish acute and chronic phases [13]. The chronically *T*. *cruzi*-infected C57BL/6 and C3H/He mice represent models of mild and severe CCC, respectively, paralleled to the degree of immunological abnormalities [22]. Sex- and age-matched noninfected (NI) controls were analyzed in parallel.

### PTX treatment

Chronically *T*. *cruzi*-infected C57BL/6 mice showing signs of CCC were intraperitoneally injected with saline (BioManguinhos/Fiocruz, Brazil) containing PTX (Trental, Sanofi-Aventis, Brazil) (20 mg/kg) or vehicle daily from 120 to 150 days postinfection (dpi).

### Reagents and antibodies

For immunohistochemical staining (IHC), the polyclonal antibody recognizing *T*. *cruzi* antigens and supernatants containing anti-mouse CD8a (clone 53–6.7) and anti-mouse CD4 (clone GK1.5) were produced in our laboratory (LBI/IOC-Fiocruz, Rio de Janeiro, RJ, Brazil). Other antibodies included an anti-F4/80 polyclonal antibody (Caltag, USA); biotinylated rabbit anti-goat IgG cocktail (KPL, USA); polyclonal rabbit anti-connexin 43 (Cx43) (Sigma-Aldrich, USA), polyclonal rabbit anti-mouse FN (Gibco-BRL, USA), biotinylated anti-mouse CD54 (intercellular cell adhesion molecule-1, ICAM-1, BD Pharmingen, USA), biotinylated anti-rat immunoglobulin (DAKO, Denmark) and biotinylated anti-rabbit immunoglobulin and peroxidase-streptavidin complex (Amersham, UK). Monoclonal antibodies anti-mouse Pfn (CB5.4, Alexis Biochemicals, USA) and anti-IFNγ (R4–6A2, BD PharMingen, USA) produced in rat were also used in IHS. For flow cytometry studies, PE-Cy7-anti-mouse TCRαβ (clone H57–597), APC-conjugated anti-mouse CD8a (clone 53–6.7), FITC-anti-CD4 (GK1.5), PE-rat anti-mouse TNF (clone MP6-XT22), PerCP-anti-CD4 (clone GK1.5), FITC- conjugated anti-Pfn (11B11) and PECy-7-conjugated anti-IFNγ (clone XMG1.2) were purchased from BD Pharmingen (USA). PE-conjugated anti-CD107a (clone eBIO1D4B) was obtained from eBioscience. Anti-TNF receptor (TNFR)1 (TNFR1/p55/CD120a; clone 55R-286) conjugated to PE was purchased from BioLegend (USA). Appropriate controls were prepared by replacing the primary antibodies with the corresponding serum, purified immunoglobulin or isotype. All antibodies and reagents were used according to the manufacturers’ instructions.

### Flow cytometry analysis

Spleens were minced and the red blood cells were removed using lysis buffer (Sigma-Aldrich, USA). In a set of experiments, peripheral blood was also collected, as previously described [9]. The splenocytes and blood cells were labeled, events were acquired with a CyAn-ADP (Beckman Coulter, USA) and the data were analyzed with the Summit v.4.3 Build 2445 program (Dako, USA) as described elsewhere [9].

### IFNγ enzyme-linked immunospot (ELISpot) assay

The ELISpot assay for the enumeration of IFNγ-producing cells was performed in triplicate as previously described [24]. Plates were coated with anti-mouse IFNγ (clone R4–6A2; BD PharMingen, USA) antibody diluted in PBS (5 μg/mL). Antigen-presenting cells were primed for 30 minutes at 37°C with total frozen extracts of epimastigote forms (Y strain) and amastigote surface protein 2 (ASP2) H-2K^b^-restricted VNHRFTLV peptide [25]. After incubation, the freshly isolated splenocytes from experimental mice were seeded at 5 x 10^5^ cells/well and incubated for 20 hours at 37°C and 5% CO_2_. Biotin-conjugated anti-mouse IFNγ antibody (clone XMG1.2; BD PharMingen, USA) was used to detect the captured cytokines. Spots were revealed after incubation of the samples with a solution of alkaline phosphatase-labeled streptavidin (BD PharMingen, USA) and a solution of NBT and BCIP (Sigma-Aldrich, USA) in Tris buffer (0.9% NaCl, 1% MgCl_2_, 1.2% Tris in H_2_O). The mean number of spots, in triplicate wells, was determined for each experimental condition. The number of specific IFNγ-secreting T-cells was calculated by estimating the stimulated spot count/10^6^ cells using a CTL OHImmunoSpot A3 Analyzer (USA).

### Detection of cytokines in the serum

A mouse cytometric bead array (CBA) Inflammation Kit (Becton & Dickinson, USA) was used to quantify cytokines in the serum according to the manufacturer’s instructions. The fluorescence produced by the CBA beads was measured with a FACSCalibur instrument (Becton Dickinson, USA) and analyzed using FCAP Array software. Standard curves (1 pg/mL to 100 ng/mL) were generated in parallel. This method consistently detected concentrations above 10 pg/mL.

### Real-time quantitative PCR

For real-time quantitative RT-PCR (RT-qPCR), the hearts were harvested, washed to remove blood clots, weighed and frozen in RNAlater (Life Technologies, USA). Total RNA (for gene expression studies) and DNA (for parasite detection) were extracted from the same sample using TRI-Reagent (Sigma-Aldrich, USA). For detection of TNF mRNA, the reverse transcriptase reactions were performed using a SuperScript III First Strand Synthesis Kit, and RT-qPCR was performed using TaqMan gene expression assays for TNF (# Mm00443258-m1) and the endogenous housekeeping control genes glyceraldehyde 3-phosphate dehydrogenase (GAPDH) (# Mm99999915-g1) and β actin (# Mm00607939-s1), purchased from Life Technologies (USA). Reactions were performed in duplicate according to manufacturer’s instruction, using cDNA template obtained from 2μg RNA. The conditions for the PCR were as follows: 95°C for 10 minutes, followed by 40 cycles at 95°C for 15 seconds and 60°C for 1 minute. Relative quantification of target gene levels was performed using the ΔΔCt method [26]. RT-qPCR data were normalized by the housekeeping genes GAPDH and β actin mRNA, using the Expression Suite Software V1.0.3 (Life Technologies, USA) and fold increase was determined in comparison with NI controls. For parasite detection 5 μL of purified DNA was analyzed by real time quantitative PCR (qPCR) using TaqMan system, with primers Cruzi 1 (5'-AST CGG CTG ATC GTT TTC GA-3'), Cruzi 2 (5'-AAT TCC TCC AAG CAG CGG ATA-3') and probe Cruzi 3: 6FAM-CACACACTGGACACCAA-MGB) targeting the *T*. *cruzi* nuclear satellite DNA, as previously [27]. As an internal amplification control, the TaqMan assay targeting mice glyceraldehyde 3-phosphate dehydrogenase (GAPDH) (# Mm99999915-g1, Life Technologies, USA) was used. Parasite load quantification was estimated by absolute quantification, following normalization by heart sample weight. The standard curve for the absolute quantification was generated by a 1:10 serial dilution of DNA extracted from *the* Colombian strain epimastigote culture stocks, ranging from 10^6^ to 0.5 parasite equivalents.

### *In vitro Trypanosoma cruzi* treatment with pentoxifylline

In a flat bottom 96-well plate (Corning, Inc, USA) were distributed 100 μL of RPMI containing 5 x 10^6^ bt/well of the *T*. *cruzi*. On parasites, were added 100 μL of different concentrations of PTX (0.3, 1, 3, 10, 30, 100 and 300 μg/mL), the positive (10 μM of the trypanocidal drug benznidazole) and negative (injection grade saline, Biomanguinhos/Fiocruz, Brazil) controls. After incubation for 24 hours at 37° C in an incubator containing constant tension of 5% CO_2_ (Shell Lab, USA), the number of parasites in the different treatment conditions was counted in a Neubauer chamber.

### Immunohistochemistry

Eight to fifteen *T*. *cruzi*-infected and three to five NI animals were euthanized under anesthesia at 120 or 150 dpi and the hearts were removed, embedded in the tissue-freezing medium Tissue-Tek (Miles Laboratories, USA) and stored in liquid nitrogen. The phenotypes of the inflammatory cells colonizing the heart tissue and the *T*. *cruzi* parasitism were characterized and analyzed as previously described [9]. The ICAM-1-, FN- and Cx43-positive areas in 25 fields (12.5 mm^2^) per section (3 sections per heart) were evaluated with a digital morphometric apparatus. The images were digitized using a color view XS digital video camera adapted to a Zeiss microscope and analyzed with AnalySIS AUTO Software (Soft Imaging System, USA). According to the analyzed parameter, the data are shown as percent of positive area in the heart, as distance (μm) between stained gap junctions or as numbers of parasite nests or cells per 100 microscopic fields of view (400 X).

### Detection of cardiac muscle creatine-kinase isoform

The activity of the creatine kinase cardiac MB isoenzyme (CK-MB) was measured using a commercial CK-MB Liquiform kit (Labtest, Brazil) according to the manufacturer’s recommendations as previously adapted for mouse samples [9].

### Electrocardiogram (ECG) registers

Mice were tranquilized with diazepam (10 mg/kg) and transducers were placed subcutaneously (DII). The traces were recorded for 2 minutes using a digital Power Lab 2/20 system connected to a bio-amplifier at 2 mV for 1 second (PanLab Instruments, Spain). The filters were standardized to between 0.1 and 100 Hz and the traces were analyzed using Scope software for Windows V3.6.10 (PanLab Instruments, Spain). The ECG parameters were analyzed as previously described [9].

### Transthoracic echocardiography

For analysis of cardiac function through echocardiography mice were anesthetized with 1.5% isoflurane gas in oxygen with flow 1L/minute, trichotomized in precordial region and examined with a Vevo 770 (Visual Sonics, Canada) coupled to a 30 MHz transducer. Cardiac geometry was made using two dimensional mode images acquired for measurement of internal area of heart cavities (right and left ventricles). M-mode images showed left ventricular (LV) muscle thickness was used for measurement LV mass. Heart and LV hypertrophy were measured by the ratios of heart weight (HW) and LV mass to body weight (BW), respectively. Left ventricular ejection fraction (LVEF) was determined using Simpson’s method and left and right ventricular (LV and RV) areas were obtained in B-mode using a short axis view at the level of the papillary muscles.

### Statistical analysis

Data are expressed as mean ± SD. Analysis was performed using GraphPrism (GraphPad, USA). Comparison between groups was carried out by analysis of variance (ANOVA) followed by Bonferroni´s post-test or t-Student test when indicated. Differences were considered statistically significant when *p<*0.05.

## Results

### PTX does not modulate TNF production but decreases TNF receptors expression in chronic *T*. *cruzi* infection

To determine the effect of PTX on immune response in chronic experimental Chagas’ heart disease, PTX administration to C57BL/6 mice infected with the Colombian *T*. *cruzi* strain was initiated at 120 dpi (S1A Fig.). At this time-point, CD8-enriched myocarditis, splenomegaly, immune abnormalities, cytokine unbalance, electrical alterations and heart injury are already installed [9,15,22,28]. At 150 dpi, all PTX-treated infected mice were alive (S1B Fig.). Low parasitemia was detected in chronically infected mice administered with saline or PTX (6.8 ± 2.2 x 10^4^ parasites /mL in saline-injected *vs* 6.4 ± 4.6 x 10^4^ parasites /mL in PTX-treated; *p*>0.05). Further, PTX therapy had no effect on body weight (22 ± 1.3 g in NI controls; 21 ± 2.5 g in saline-injected *vs* 20.1 ± 0.8 g in PTX-treated infected mice; *p*>0.05). In comparison with sex- and age-matched NI controls, chronically *T*. *cruzi*-infected C57BL/6 mice presented splenomegaly (*p*<0.001), which is significantly (*p*<0.05) reversed by PTX therapy (S1C Fig.). Chronically *T*. *cruzi*-infected C57BL/6 mice have increased levels of TNF in the serum (S2A Fig.) and TNF mRNA in the heart tissue (S2B Fig.), corroborating previous data [15,22,28]. Further, at 150 dpi C57BL/6 mice have increased frequency of TNF-producing CD8^+^ T-cells in spleen (S2C Fig.). One of the proposed beneficial effects of PTX is its capacity of modulate TNF production [16]. However, PTX treatment from 120 to 150 dpi had no effect on *T*. *cruzi*-induced high TNF levels in the serum (S2A Fig.), TNF mRNA overexpression in the heart tissue (S2B Fig.) and TNF expression by CD8^+^ T-cells in spleen (S2C Fig.).

TNF signals via TNFR1/p55/CD120a and TNFR2/p75/CD120b [29]. In chronically *T*. *cruzi*-infected saline-injected C57BL/6 mice, there was a remarkable increase in the frequency of CD8^+^ TNFR1^+^ and CD8^+^ TNFR2^+^ T-cells in the spleen (S2D Fig.). PTX was previously shown to downmodulate TNFR1 expression by hepatic cells [30]. Importantly, PTX therapy (from 120 to 150 dpi) completely abrogated the elevated frequency of TNFR1^+^ CD8^+^ T-cells. Nevertheless, PTX therapy only partially reduced the high frequency of TNFR2-bearing CD8^+^ T-cells detected in chronically infected mice (S2D Fig.). These data support that PTX was selectively active in chronic experimental CD.

### PTX treatment rescues the downregulation of TCR on CD8^+^ cells in chronically *T*. *cruzi*-infected mice

**Fig 1:**
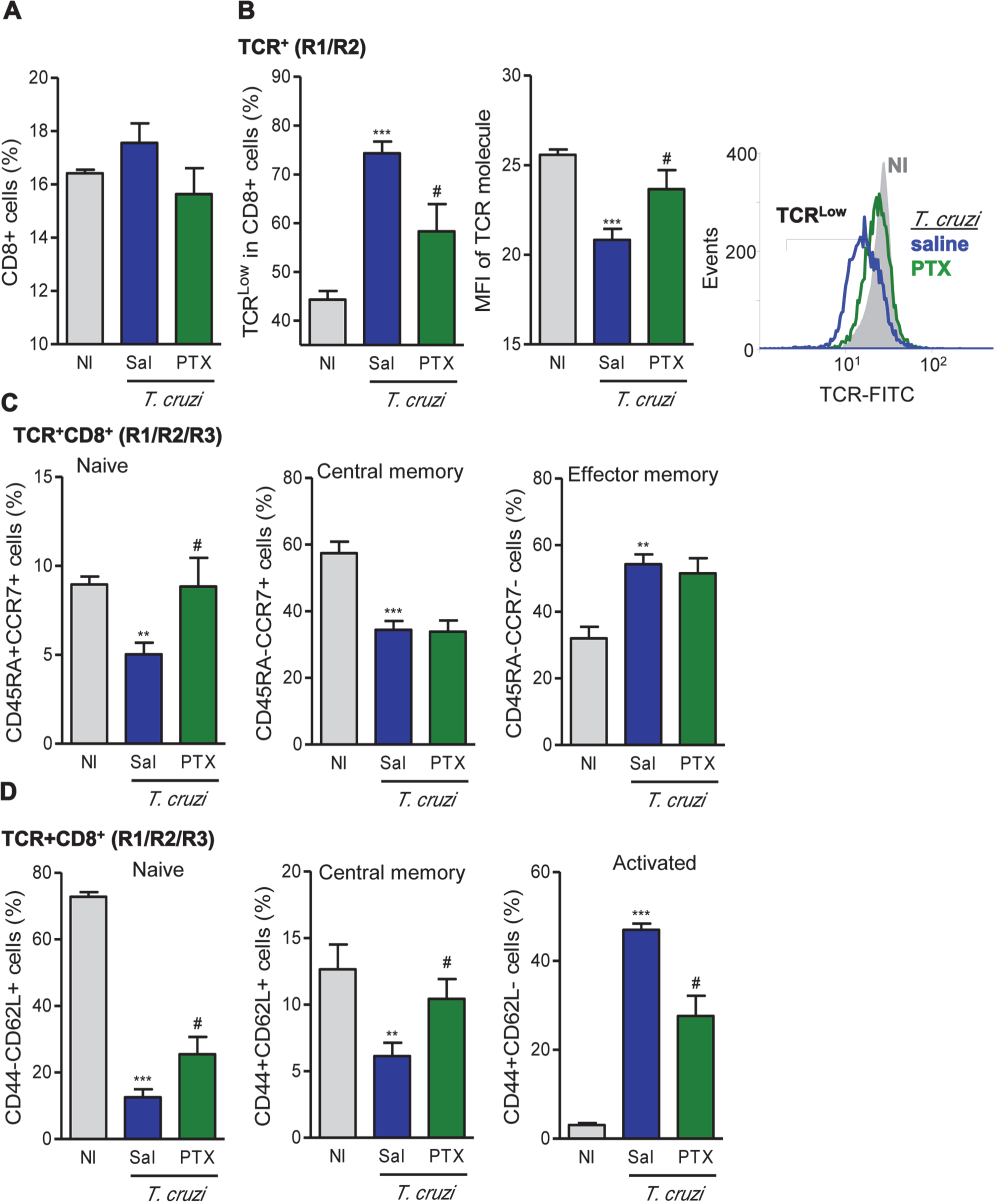
PTX therapy rescued TCR expression and influenced naïve/memory/activation phenotypes of CD8^+^ T-cells. (A) Flow cytometry analysis of CD8^+^ T-cells in the spleen. (B) Frequency of TCRαβ^Low^ cells and mean fluorescence intensity (MFI) of TCR in CD8^+^ T-cells. (C) Frequencies of splenic TCR^+^CD8^+^ cells expressing CD45RA^+^CCR7^+^ (naïve), CD45RA^-^CCR7^+^ (central memory) and CD45RA^-^CCR7^-^ (effector memory). (D) Frequencies of CD44^-^CD62L^+^ (naïve), CD44^+^CD62L^+^ (central memory) and CD44^+^CD62L^-^ (activated) CD8^+^ T-cells. The results represent three to five mice per experimental group in three independent experiments. ** *p<*0.01 and ***p<0.001, saline-injected *T*. *cruzi*-infected mice compared with noninfected (NI) controls. ^#^ *p<*0.05, saline-injected compared with PTX-treated *T*. *cruzi*-infected mice.

At 150 dpi, although a significant reduction in splenomegaly was noticed in PTX-treated mice (S1C Fig.), similar frequencies of CD8^+^ T-lymphocytes were detected in the spleen of NI controls and saline-injected and PTX-treated chronically infected mice (Fig. 1A). Further, CD8^+^ T-cells also express similar density of the CD8 molecule on cell surface in the studied groups (MFI in NI: 25.6 ± 0.6; saline-injected *T*. *cruzi*-infected: 20.8 ± 1.5; PTX-treated *T*. *cruzi*-infected: 23.7 ± 2.6). However, compared with NI controls, a considerable part of the splenic CD8^+^ cells of chronically Colombian-infected mice expressed TCRαβ^Low^ (Fig. 1B; *p*<0.001), corroborating previous data in a distinct model of chronic CD [6]. Importantly, PTX treatment significantly rescued the downregulation of TCR expression in CD8^+^ T-cells of chronically *T*. *cruzi*-infected mice (Fig. 1B), considering both frequency of TCRαβ^Low^ population (*p*<0.05) and density of TCRαβ on cell surface (*p*<0.05).

### Memory and activation profiles of CD8^+^ T-Cells are modified by PTX treatment in chronic infection

Next, we investigated whether PTX therapy influenced the expression of markers of naïve/memory/activation phenotypes of CD8^+^ T-cells during chronic *T*. *cruzi* infection using CD45, a tyrosine phosphatase essential for T-cell activation, and the expression of CCR7, a chemokine receptor associated with the homing of T-cells to lymph nodes [31]. Compared with NI controls, splenic TCR^+^CD8^+^ T-cells of chronically infected mice displayed low frequencies of CD45RA^+^CCR7^+^ (naïve; *p*<0.01) cells and CD45RA^-^CCR7^+^ (central memory; *p*<0.001) cells, but showed increased frequency of CD45RA^-^CCR7^-^ (effector memory; *p*<0.01) cells (Fig. 1C). PTX treatment restored the frequencies of the minor CD45RA^+^CCR7^+^ T-cell subset (*p*<0.05), but did not interfere with the proportions of the major CD45RA^-^CCR7^+^ and CD45RA^-^CCR7^-^ CD8^+^ T-cell populations (Fig. 1C). In an attempt to further dissect the immunoregulatory mechanism of PTX in chronic experimental CD, we analyzed the frequencies of naïve, memory and activated T-cells studying the expression of CD44, a chondroitin sulfate proteoglycan receptor associated with cell migration to peripheral tissues, and CD62L, a marker of T-cell homing to lymph nodes [31]. In chronic *T*. *cruzi* infection, there was a remarkable decrease in the frequencies of CD44^-^CD62L^+^ naïve (*p*<0.001) and CD44^+^CD62L^+^ central memory (*p*<0.01) but an increase in the frequency of CD44^+^CD62L^-^ (*p*<0.001) CD8^+^ T-cells (Fig. 1D), in comparison with age-matched NI mice. Notably, PTX therapy partially restored these drastic alterations, significantly (*p*<0.05) increased the frequencies of CD44^-^CD62L^+^ and CD44^+^CD62L^+^ cells and decreased the proportion of CD44^+^CD62L^-^ CD8^+^ T-cells in the spleen (Fig. 1D). However, there were no changes in the frequencies of CD8^+^ CD44^+^CD62L^-^ (46.3 ± 3.3% in saline-injected *vs* 51.9 ± 5.5% in PTX-treated *T*. *cruzi*-infected mice; *p*>0.05) and CD8^+^ CD44^+^CD62L^+^ (15.4 ± 3.3% in saline-injected *vs* 10.7 ± 3.4% in PTX-treated *T*. *cruzi*-infected mice; *p*>0.05) T-cells in the blood of C57BL/6 mice.

### PTX administration sustained IFNγ^+^ cells systemically and in the heart tissue, but reduced the number of cytotoxic Pfn^+^ cells in the cardiac tissue

**Fig 2:**
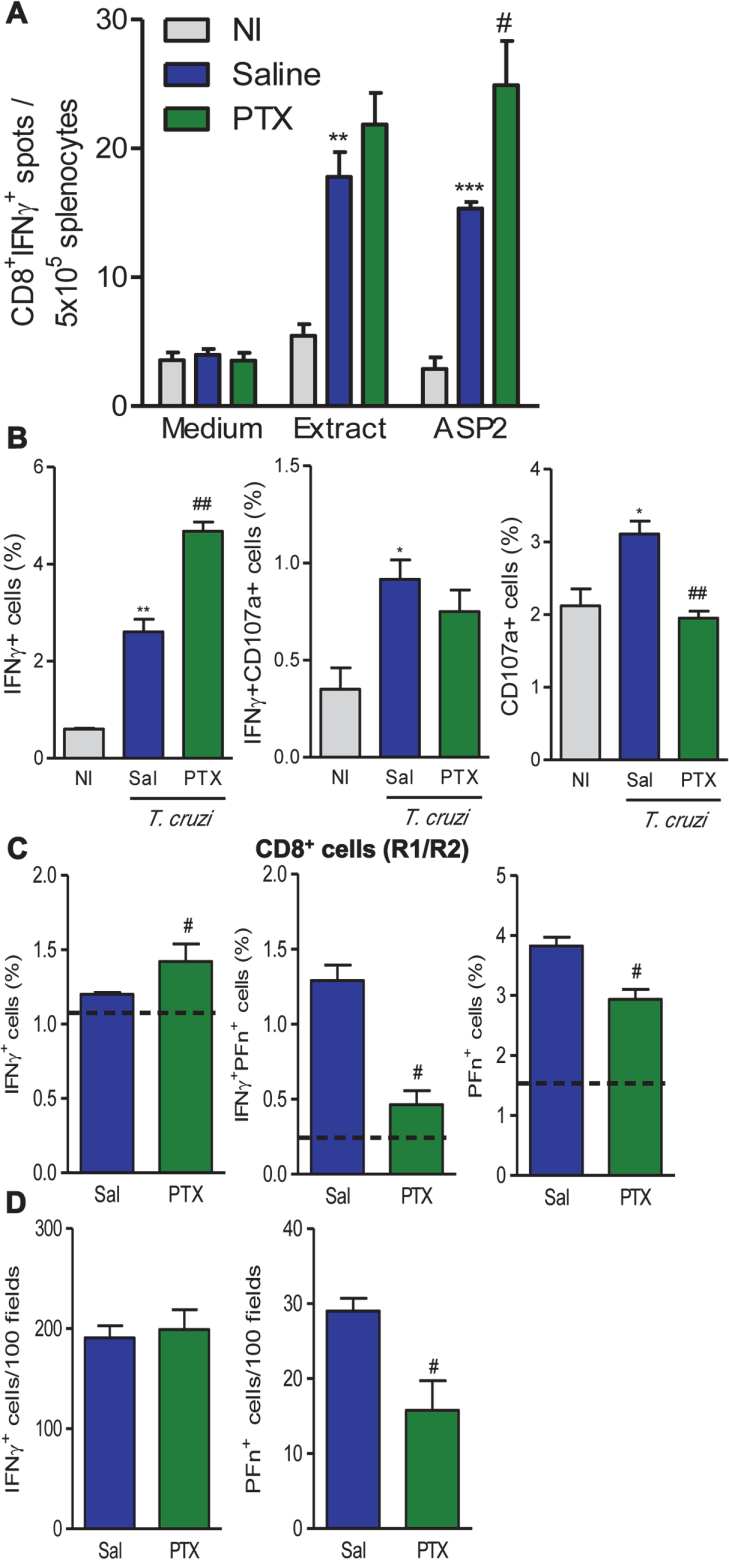
PTX influenced the balance of inflammatory and cytotoxic profiles systemically and in the heart tissue. (A) ELISpot assay identifying the functional capacity of IFNγ-producing CD8^+^ T-cells recognizing *T*. *cruzi* crude extracts and the H-2K^b^-restricted ASP2 (VNHRFTLV) immunodominant peptide. (B) Frequencies of IFNγ^+^, IFNγ^+^CD107a^+^ and CD107a^+^ CD8^+^T-cells in spleen. (C) Frequencies of IFNγ^+^, IFNγ^+^PFn^+^ and PFn^+^ CD8^+^T-cells in spleen. Dotted lines show mean frequencies in noninfected (NI) controls. (D) Immunohistochemistry for detection of IFNγ^+^ and Pfn^+^ cells in the heart tissue. The results represent three to five mice per experimental group in three independent experiments. * *p<*0.05, ** *p<*0.01 and *** *p<*0.001, saline-injected *T*. *cruzi*-infected mice compared with NI controls. ^#^ *p<*0.05 and ^##^ *p<*0.01, saline-injected compared with PTX-treated *T*. *cruzi*-infected mice.

Next, we explored the potential influence of PTX on the effector function of *T*. *cruzi*-specific total and CD8^+^ T-cells of chronically *T*. *cruzi*-infected C57BL/6 mice. Using ELISpot assay, we detected an increased (*p*<0.01) number of T-cells producing IFNγ after recognition of crude *T*. *cruzi* antigens (epimastigote extracts) in chronically infected mice. Also, the number of IFNγ-producing CD8^+^ T-cells specific for the immunodominant H-2K^b^-restricted ASP2 VNHRFTLV peptide was increased (*p*<0.001) (Fig. 2A). PTX therapy did not alter the number of IFNγ-producing cells among splenocytes recognizing crude *T*. *cruzi* antigens, but upregulated (*p*<0.05) the number of IFNγ-producing ASP2-specific CD8^+^ T-cells (Fig. 2A). Having in mind the different CD8^+^ T-cell phenotypes, we evaluated the potential cytotoxic activity by CD8^+^ splenocytes of chronically infected mice, studying the expression of CD107a, a marker for T-cell degranulation [32], and inflammatory potential, studying intracellular IFNγ expression. At 150 dpi, in comparison with NI controls, there was a significant increase in the frequencies of IFNγ^+^ (*p*<0.01) and IFNγ^+^CD107a^+^ and CD107a^+^ (*p*<0.05) CD8^+^ T-cells in saline-injected infected mice (Fig. 2B and S3 Fig.). PTX therapy significantly increased the frequency of IFNγ^+^ CD8^+^ T-cells (*p*<0.01) and reduced the frequency of CD107a^+^ (*p*<0.01) CD8^+^T-cells (Fig. 2B and S3 Fig.). Considering the antagonistic roles for IFNγ^+^ and Pfn^+^ CD8^+^ T-cells in *T*. *cruzi* infection [9], we studied the effect of PTX on IFNγ and Pfn expression by CD8^+^ T-cells. PTX administration to chronically infected mice increased (*p*<0.05) the frequency of IFNγ^+^ cells, but reduced (*p*<0.05) the frequencies of IFNγ^+^Pfn^+^ and Pfn^+^ CD8^+^ T-cells (Fig. 2C). To investigate whether PTX effects on inflammatory IFNγ and cytotoxic Pfn^+^ cells were restricted to splenic compartment, we analyzed the numbers of IFNγ^+^ and Pfn^+^ inflammatory cells invading the heart tissue of chronically infected C57BL/6 mice. Saline-injected and PTX-treated chronically infected mice had similar numbers of IFNγ^+^ cells in the heart tissue (Fig. 2D). In comparison with saline injection, PTX therapy reduced (*p*<0.05) the number of inflammatory Pfn^+^ cells infiltrating the cardiac tissue of chronically infected mice (Fig. 2D).

### PTX modulates the activation of blood vessel endothelial cells and reduced the number of inflammatory cells invading the heart tissue

**Fig 3:**
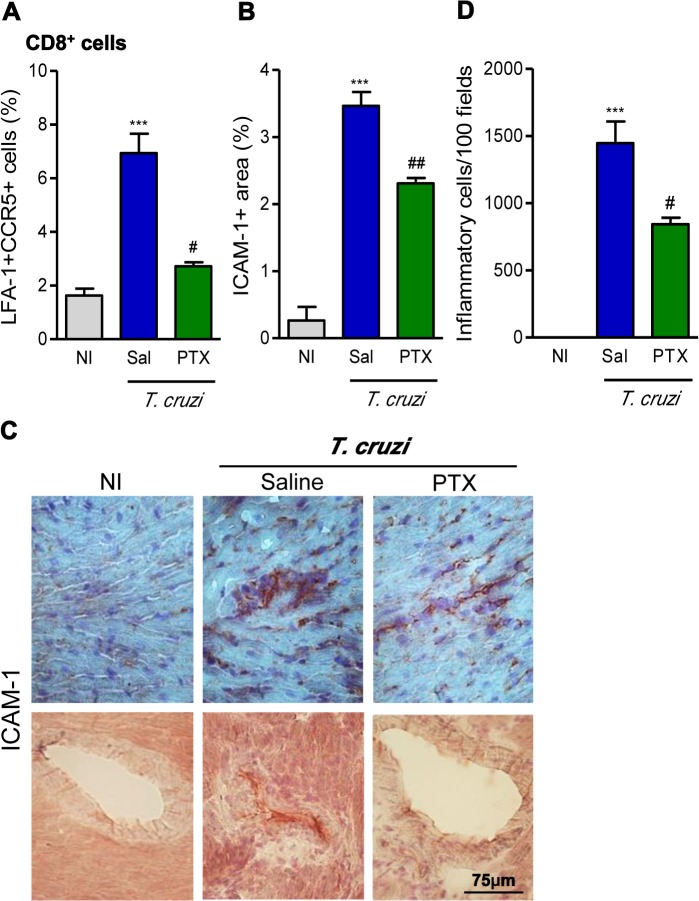
PTX modulated the expression of molecules involved in cell migration and reduced heart inflammation. (A) Frequency of CCR5^+^LFA-1^+^ cells among CD8^+^ T-cells in spleen. (B) Quantification of the ICAM-1^+^ area (%) detected by immunohistochemistry in the heart tissue. (C) Illustrative pictures of immunohistochemistry staining for detection of ICAM-1 in the heart tissue and blood vessels at 150 dpi. (D) Summary of quantitative data of immunohistochemistry staining for inflammatory cells in the heart tissue. The results represent three to five mice per experimental group. *** *p<*0.001, saline-injected *T*. *cruzi*-infected mice compared with noninfected (NI) controls. ^#^ *p<*0.05 and ^##^ *p<*0.01, saline-injected compared with PTX-treated *T*. *cruzi*-infected mice.

Our previous data support that the formation of CD8-enriched chagasic myocarditis involves CCR1/CCR5-mediated cell migration [33,34]. Further, the CCR5 receptor and the cell adhesion molecule LFA-1 are co-expressed by peripheral blood mononuclear cells enabling them to migrate to heart tissue [33,35]. Here we described an increased (*p*<0.001) frequency of CD8^+^ T-cells co-expressing LFA-1 and CCR5 among CD8^+^ T-cells in chronically infected mice. Importantly, PTX treatment led to a significant reduction in the frequency of splenic LFA-1^+^CCR5^+^ CD8^+^ T-cells (Fig. 3A), when compared with saline injection. Moreover, a significant reduction in the frequency LFA-1^+^CCR5^+^ was also observed among circulating CD8^+^ T-cells after PTX therapy (3.14 ± 1.0% in PTX-treated *vs* 5.75 ± 2.2% in saline-injected *T*. *cruzi*-infected mice; *p*<0.05). Next, we analyzed the expression of ICAM-1, the LFA-1 ligand, on the cardiac endothelial cells [36]. ICAM-1 is upregulated (*p*<0.001) in the endothelial cells of heart blood vessels and cardiomyocytes of chronically *T*. *cruzi*-infected C57BL/6 mice (Fig. 3B and Fig. 3C). After PTX therapy, reduction (*p*<0.01) in ICAM-1 expression was noticed in cardiomyocytes and inflammatory cells infiltrating the heart tissue (Fig. 3C—upper panel) and, particularly, in the blood vessel endothelial cells (Fig. 3C—bottom panel). ICAM-1^+^ blood vessels with perivascular cuffs with several layers of inflammatory cells were commonly found in the heart of saline-injected but absent in PTX-treated *T*. *cruzi*-infected mice (Fig. 3B and Fig. 3C). At 150 dpi, the Colombian-infected C57BL/6 mice present myocarditis (Fig. 3D), mainly composed of CD8^+^ T-cells, corroborating previous findings [9]. Interestingly, the short term PTX therapy reduced (*p*<0.05) the intensity of the chronic *T*. *cruzi*-induced myocarditis (Fig. 3D).

### PTX does not interfere with *Trypanosoma cruzi* growth control

The pathogenesis of Chagas’ heart disease is, at least in part, accounted to parasite persistence [2,37]. To bring further mechanistic insights into the beneficial effects of PTX in chronic infection, we analyzed a putative effect of PTX directly on *T*. *cruzi* trypomastigote forms and on heart parasitism. Contrasting with a significant (*p*<0.001) effect of the trypanocidal drug Bz (positive control), PTX showed no direct action on the survival of the trypomastigote forms of the parasite, in an *in vitro* assay (S4A Fig.). Meanwhile, PTX did not interfere with parasite control in chronically infected C57BL/6 mice, as rare *T*. *cruzi* amastigote nests (S4B Fig.) and low numbers of parasite DNA copies (S4C Fig.) were similarly (*p*>0.05) detected in the heart tissue of saline-injected and PTX-treated mice, at 150 dpi.

### PTX treatment ameliorates *T*. *cruzi*-induced heart injury

**Fig 4:**
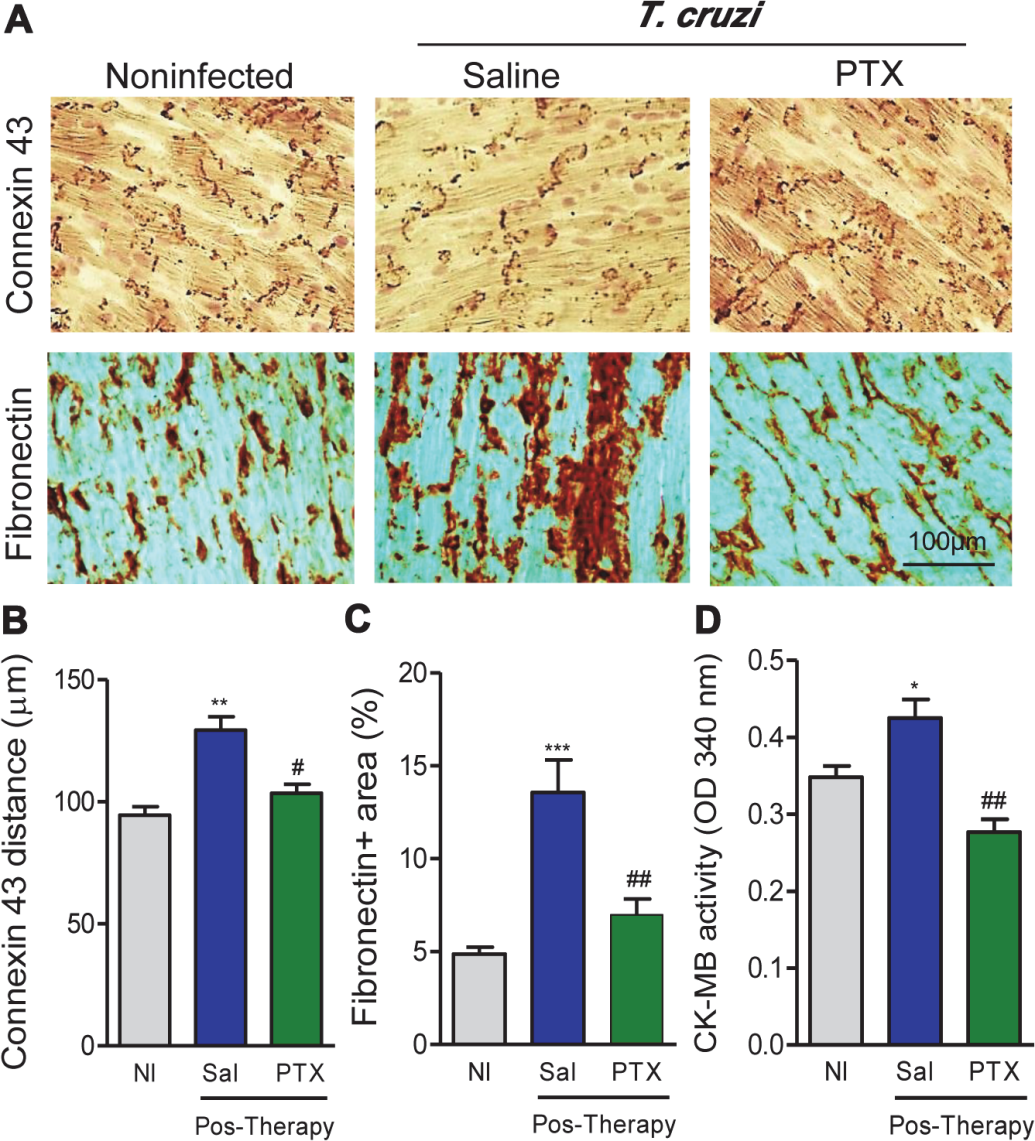
PTX therapy reduced heart injury in chronic *Trypanosoma cruzi* infection. (A) Representative sections of Cx43 gap junction expression and fibronectin-stained areas detected by immunohistochemistry in the heart tissue at 150 dpi. (B) Quantification of the distances of Cx43-containing gap junction in the heart tissue. (C) fibronectin^+^ area (%) detected by immunohistochemistry in the heart tissue. (D) Evaluation of CK-MB activity in the serum at 150 dpi. The results represent three to five mice per experimental group in three independent experiments. * *p<*0.05, ** *p<*0.01 and *** *p<*0.001, saline-injected *T*. *cruzi*-infected mice compared with noninfected (NI) controls. ^#^ *p<*0.05 and ^##^ *p<*0.01, saline-injected compared with PTX-treated *T*. *cruzi*-infected mice.

All the beneficial effects of PTX on the unbalanced immune response of chronically infected mice encouraged us to investigate the effects of PTX therapy on *T*. *cruzi*-induced chronic heart injuries. Chronically *T*. *cruzi*-infected mice showed a significant reduction (*p*<0.01) in Cx43 expression in the intercalary disc of myocardial cells, seen as increased distance of the Cx43-bearing gap junction plaques (Fig. 4A and Fig. 4B). Further, chronically infected mice presented FN overdeposition in the heart tissue (*p*<0.001, Fig. 4A and Fig. 4C), and CK-MB activity levels in the serum (*p*<0.05, Fig. 4D), compared with age- and sex-matched NI controls. In comparison with saline injection, PTX therapy ameliorated heart tissue injuries, improving Cx43 expression (*p*<0.05, Fig. 4A and Fig. 4B), decreasing FN overexpression (*p*<0.01, Fig. 4A and Fig. 4C) and reducing CK-M activity in the serum (*p*<0.01, Fig. 4D). Thus, PTX therapy hampered the progression of heart injury in chronically *T*. *cruzi-*infected C57BL/6 mice. Moreover, considering that significant increase in CK-MB activity is already detected in *T*. *cruzi*-infected C57BL/6 mice at 120 dpi [9], our data support that PTX therapy reversed cardiomyocyte injury.

### PTX treatment prevents the progression of ECG and ECO abnormalities

**Fig 5:**
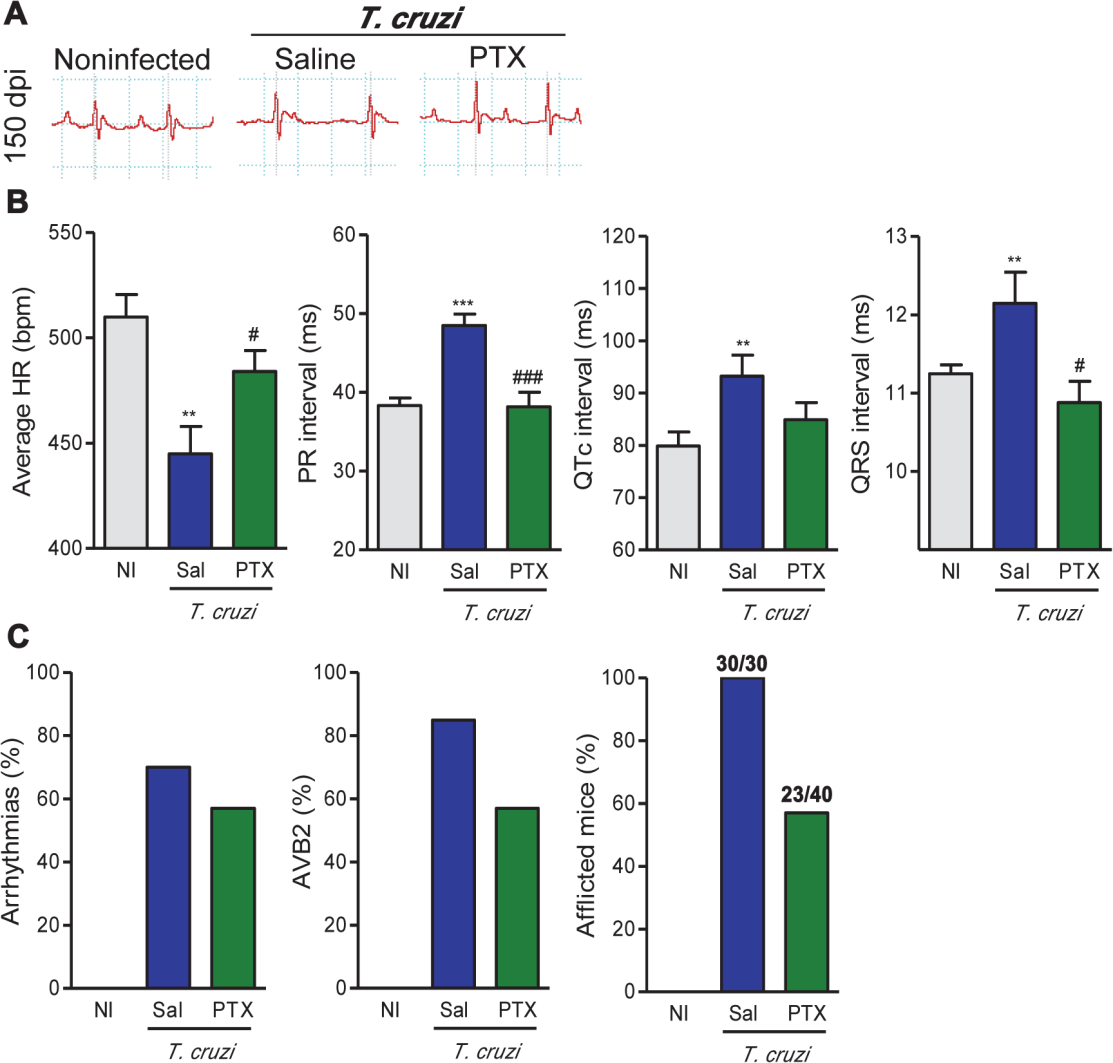
PTX therapy ameliorated ECG alterations in chronic experimental Chagas’ heart disease. (A) Representative ECG register segments of sex- and age-matched noninfected (NI) controls and infected mice (150 dpi) injected with saline or PTX. (B) Group data for ECG records showing the heart rate (beats per minute, bpm), variation in PR, QTc and QRS intervals. (C) Summary of the group data from NI and infected mice showing the frequencies of mice afflicted by arrhythmias (ART), second degree atrio-ventricular block (AVB2) and any ECG alterations in three independent experiments. ** *p<*0.01 and *** *p<*0.001, saline-injected *T*. *cruzi*-infected mice compared with NI controls. ^#^ *p<*0.05 and ^###^ *p<*0.001, saline-injected compared with PTX-treated *T*. *cruzi*-infected mice.

After the demonstration that PTX restored major immunological abnormalities believed to be associated with the severity of Chagas’ heart disease [9,10,15,21,22], we examined the effect of PTX on electrical conduction in an experimental model of CCC. When compared with sex- and age-matched NI controls, saline-injected chronically infected C57BL/6 mice presented ECG alterations including prolonged P wave, PR interval and QRST complex (Fig. 5A). At 150 dpi, PTX-treated mice improved ECG alterations, compared with saline-injected mice (Fig. 5A). Notably, PTX had beneficial effects on heart rate (*p*<0.05), PR (*p*<0.001) and QRS (*p*<0.05) intervals in comparison with saline-injected animals (Fig. 5B). Actually, PTX therapy reduced the proportion of mice afflicted by arrhythmias (ART), second-degree atrio-ventricular block (AVB2) and other ECG abnormalities (Fig. 5C). At 120 dpi, ECG abnormalities are already detected in *T*. *cruzi*-infected C57BL/6 mice [9]; hence, our data support that PTX therapy reversed ECG alterations.

In parallel experiments, parasitemia, heart parasitism and inflammation were higher in Colombian-infected C3H/He compared with C57BL/6 mice, at 120 dpi [22]. In the model of severe infection (C3H/He), PTX therapy also ameliorated the expression of the biomarkers of heart injury Cx43 loss and FN deposition in the heart tissue, CK-MB activity levels in the serum (*p*<0.01, S5A Fig. and S5B Fig.) and ECG abnormalities (*p*<0.05, S5C Fig. and S5D Fig.). At 150dpi, the increased P duration, QTc and QRS intervals (*p*<0.05, S5C Fig.) and the proportions of mice afflicted by ECG abnormalities were diminished by PTX therapy (S5D Fig.).

**Fig 6:**
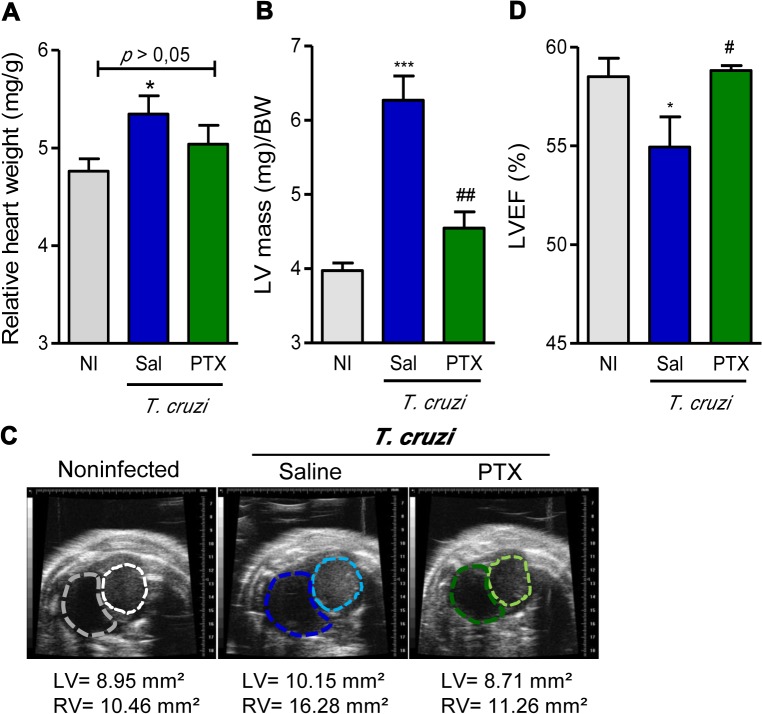
PTX treatment improved heart dysfunction in chronic experimental Chagas’ heart disease. (A) Heart weight/body weight (HW/BW) coefficient. (B) Left ventricular mass (LVM). (C) Representative echocardiography images of ventricular areas. (D) Percentage of left ventricular ejection fraction (LVEF). * *p<*0.05 and *** *p<*0.001, saline-injected *T*. *cruzi*-infected mice compared with noninfected (NI) controls. The results represent eight to thirteen mice per experimental group. ^#^ *p<*0.05 and ^##^ *p<*0.01, saline-injected compared with PTX-treated *T*. *cruzi*-infected mice.

At 150 dpi, all analyzed groups of C57BL/6 mice showed similar body weight (NI: 23.2 ± 1.3; saline-injected *T*. *cruzi*-infected: 20.6 ± 1.3; PTX-treated *T*. *cruzi*-infected: 22.5 ± 2.0). Chronic *T*. *cruzi* infection resulted in heart enlargement, shown as increased HW/BW ratio (Fig. 6A), corroborating our previous data [9]. The HW/BW coefficient of PTX-treated mice tends to decrease when compared with saline-injected infected mice and was similar to the HW/BW coefficient of sex- and age-matched NI controls (*p>*0.05; Fig. 6A). To assess whether chronic *T*. *cruzi* infection affects heart geometry and function, as well as the impact of PTX administration, all chronically infected mice underwent echocardiographic evaluation. At 150 dpi, compared with NI C57BL/6 controls, infected mice showed alterations in heart geometry, as increased LV mass (NI: 82.4 ± 2.9; saline-injected *T*. *cruzi*-infected: 100.7 ± 4.3; *p<*0.01). PTX therapy significantly reduced LV mass (PTX-treated *T*. *cruzi*-infected: 80.1 ± 4.3; *p<*0.01), restoring mass values akin to NI controls. The assessment of heart hypertrophy, as the ratio of LV mass to body weight, showed a remarkable LV hypertrophy (*p<*0.01) in chronically Colombian-infected mice (Fig. 6B). Importantly, PTX therapy significantly reduced the LV hypertrophy (*p<*0.01) of *T*. *cruzi*-infected mice (Fig. 6B). Additionally, compared with NI controls, saline-injected chronically infected mice showed higher right ventricular (RV) and LV areas (Fig. 6C). PTX-treated mice exhibited RV and LV areas similar to age-matched NI controls (Fig. 6C). When compared with NI age-matched controls, mice chronically infected by the Colombian strain showed significant decrease in LVEF (Fig. 6D). Notably, PTX therapy significantly (*p<*0.05) restored LVEF of chronically infected mice to values resembling NI controls (Fig. 6D).

## Discussion

Here we used PTX as a therapeutic tool in experimental CCC, exploring the effects of this immunomodulator upon key features of immunological and heart abnormalities. After PTX therapy, the overexpression of TNF remained unaltered, while TNFR1 and TNFR2 expression was reduced. PTX therapy also decreased the frequency of splenic cytotoxic (CD107a^+^ and Pfn^+^) CD8^+^ T-cells and the number of Pfn^+^ cells invading the cardiac tissue. Conversely, treatment with PTX increased the number of IFNγ-producing ASP2-specific CD8^+^ T-cells. Further, PTX-treated mice show reduced frequency of splenic and circulating LFA-1^+^CCR5^+^ CD8^+^ T-cells, decreased expression of ICAM-1 on cardiac tissue and less severe myocarditis. Importantly, PTX therapy ameliorated heart injury and dysfunction, without interfering with parasite control.

Splenomegaly is present in chronic CD patients [38]. In experimental *T*. *cruzi* infection, splenomegaly is associated with increased polyclonal T-cell activation, a hallmark of not regulated immune response [6,9, 38]. Notably, the splenomegaly seen in Colombian-infected C57BL/6 mice [9,15] was partially reversed by PTX therapy. This effect may result of the inhibitory effect of PTX on primary proliferative capacity of T-cells [39].

PTX ameliorates LVEF in patients with noninfectious heart failure in association with downmodulation of inflammatory biomarkers, such as TNF [18,19]. Elevated plasma TNF levels paralleled CCC severity in patients [21,40] and chronic experimental CD models [15,22]. During acute *T*. *cruzi* infection, PTX administration reduced the number of TNF^+^ cells in necrotic areas in the spleen [41]. Thus, a reduction in TNF expression was expected in our models of CCC subjected to PTX treatment. However, this was not the case as PTX did not affect TNF overexpression in the heart tissue or systemically (spleen and serum). The lack of effect of PTX on TNF expression in experimental CCC was not a surprise. Clinical trials assessing PTX in chronic heart failure showed no concordant action on downmodulation of TNF levels, despite clinical improvement and beneficial effects on biomarkers of heart lesion [16]. The mechanism by which PTX improves heart diseases remains unsolved [16,18,19]. TNF signals through two different receptors (TNFR1 and TNFR2) and affects diverse biological processes such as cell activation, proliferation, differentiation and survival [29]. Further, depending on the biological process TNF receptors may have opposite effects [42]. In CCC, PTX abrogated TNFR1 expression by CD8^+^ T-cells. Also, in ischemic-reperfusion hepatic injury the beneficial effect of PTX was associated with abrogation of the high expression of TNFR1 mRNA [30]. Thus, the downmodulatory effect of PTX on TNFR1 occurs independently of the trigger and target cell type. Additionally, in chronically infected mice PTX therapy partially reduced the upregulated expression of TNFR2 by CD8^+^ T-cells. Since TNFR2 receptor is involved in cell proliferation [29] and upregulated after T-cell activation [43], in reducing the frequency of TNFR2-expressing cells may reside, at least in part, the beneficial effect of PTX on splenomegaly during chronic *T*. *cruzi* infection. Therefore, akin the effect of anti-TNF therapy in CCC [15], PTX may emerge as a tool to disrupt TNF/TNFRs signaling pathway and restore immunological homeostasis and cardiac injury in experimental CCC.

CD8^+^ T-cells are the prominent inflammatory components in the cardiac tissue in Chagas’ heart disease [3,4,14], a feature reproduced in Colombian-infected mice [9,13,15,28,34]. In CD patients, CD8^+^ T-cells show abnormal activation phenotypes marked by low expression of CD8 and TCR [7]. Although in chronically Colombian-infected mice no changes in the frequency of splenic CD8^+^ T-cells and in the density of CD8 molecules on cell surface were detected, downmodulation of TCRαβ expression on CD8^+^ T-cells was noticed, corroborating previous data [6]. Importantly, in PTX-treated mice the frequency of TCR-bearing cells and the density of TCRαβ on cell membrane were restored to intensities similar to NI controls. In naïve T-cells, TCR complex is constitutively internalized and rapidly recirculate back to cell surface. However, by a molecular process not yet fully understood, antigen stimulation increases retention/degradation of TCR lowering the density of TCR on cell surface in association with reduced effector function [44, 45]. Therefore, in chronic *T*. *cruzi*-infection PTX therapy mice may interfere with the turnover of TCR and restore the capacity of CD8^+^ T-cells to respond to activation signals. This idea deserves to be explored.

In comparison with noninfected individuals, CD patients show significant increase in total effector/memory CD8^+^ T-cells (CD45RA^−^CCR7^−^), supporting a continuous stimulus by parasite antigens [7]. Similarly, increased frequency of effector/memory CD45RA^−^CCR7^−^ CD8^+^ T-cells was detected in chronically infected C57BL/6 mice. PTX did not interfere with the frequency of these cells, supporting that they are potentially prone to control invasive pathogens [31]. Conversely, PTX therapy restored the naive CD45RA^+^CCR7^+^ CD8^+^ T-cell compartment, potentially seeking homeostasis and allowing immune response to new stimuli. Here we corroborated the findings that chronic *T*. *cruzi* infection reduced the frequency of CD62L^+^ (naïve/central memory) and increased the percentage of CD44^+^CD62L^−^ (activated) cells among CD8^+^ T-cells [13,46]. Interestingly, PTX therapy decreased the frequency of CD44^+^CD62L^−^ and increased the proportion of CD44^−^CD62L^+^CD8^+^ splenocytes. Therefore, PTX interferes with cell migration and increases retention of T-cells in secondary lymphoid tissues, major sites of antigen recognition [47]. In chronic *T*. *cruzi* infection, another remarkable disturb in cell migration scenario is the increased frequency of splenic and circulating LFA-1^+^CCR5^+^ CD8^+^T-cells [9,13,34]. These cells are potentially able to invade the heart tissue, where CCR5 ligands (CCL3/MIP-1α and CCL5/RANTES) are found [13,28,34]. As PTX therapy lowered the frequencies of splenic and circulating LFA-1^+^CCR5^+^ CD8^+^ T-cells in chronically infected mice, a reduction in myocarditis intensity was expected. Indeed, PTX decreased the number of inflammatory cells invading the heart tissue. *T*. *cruzi* infection increases the expression of ICAM-1 (ligand of LFA-1) on cardiac endothelial cells and cardiomyocytes [13,48], aiding T-cell entry into the heart tissue [35]. In chronically infected mice, PTX decreased the expression of ICAM-1on cardiac tissue. PTX was also shown to downmodulate ICAM-1 in a model of acute lung injury [49]. Therefore, in chronically infected mice PTX may reduce the CCR5-mediated chemotaxis and LFA-1/ICAM-1-mediated endothelial cell/lymphocyte interaction, explaining the decreased colonization of the heart tissue by inflammatory cells. CCR5 expression is related to CCC in patients [50] and myocarditis intensity and heart injury in infected mice [33,34]. In *T*. *cruzi* infection the majority of the detrimental Pfn^+^CD8^+^ cells are LFA-1^+^CCR5^+^ [9], hence we predicted a beneficial effect of PTX in experimental CCC in association with a selective reduction of the migration of Pfn^+^ cells to heart tissue.

In experimental *T*. *cruzi* infection, antigen specific Pfn^+^CD8^+^ T-cells may play a non-beneficial role, whereas IFNγ^+^CD8^+^ T-cells may exert a protective role in heart injury [9]. In CD patients, the frequency of IFNγ-producing CD8^+^ T-cells specific for *T*. *cruzi* antigens is inversely related with disease severity [7]. Therefore, we tested the impact of PTX on specific T-cell response in chronically infected mice. Notably, PTX therapy increased the number of IFNγ-producing anti-*T*. *cruzi* VNHRFTLV ASP2 effector CD8^+^ T-cells, a population shown to protect against *T*. *cruzi* infection [51]. Previously, PTX was shown to increase T-cell memory and protective immunity against *Salmonella* infection [52]. In refractory patients, PTX combined with antimonial drug ameliorated cutaneous leishmaniasis [17], supporting the use of PTX as immunological adjuvant. In chronically *T*. *cruzi*-infected mice, PTX therapy also increased the frequency of splenic IFNγ-producing CD8^+^ T-cells and reduced the proportion of CD8^+^ T-cells expressing CD107a, a marker for T-cell degranulation and cytotoxic activity [32]. Moreover, PTX treatment decreased the frequencies of Pfn^+^ and multifunctional Pfn^+^IFNγ^+^ CD8^+^ T-cells. Based on the possible antagonistic role of CD8^+^T-cells expressing IFNγ and Pfn, [9,15], we then analyzed the influence of PTX on the composition of chronic heart inflammation. PTX-treated mice had reduced number of Pfn^+^ cells, although the number of IFNγ^+^ cells remained unaltered. The heart infiltrating Pfn^+^ cells, probably CD8^+^ T-cells acting as CTLs, are involved in tissue damage in experimental CD [9,15]. Interestingly, in CD patients with severe cardiomyopathy the presence of cells expressing granzyme A (another component of the lytic machinery of CTL CD8^+^ T-cells) in heart lesions is in accordance with concepts that involve cytolysis in pathogenesis of CCC [9,53]. Further, there is a good correlation between the numbers of IFNγ^+^ and CD8^+^ cells infiltrating the heart tissue in CD patients presenting successful parasite control [53]. Actually, PTX did not influence cardiac or systemic parasite load, reinforcing that an effective immune response, which contributes to *T*. *cruzi* control, is preserved and disconnected from factors causing heart injury [9,15,33,34].

Altogether, these findings support that PTX therapy in experimental CCC reduced the ability of CD8^+^ T-cells to migrate and invade the heart tissue, which is less permissive to lymphocyte interaction. Further, PTX diminished the frequency of activated but increased the frequency of naïve CD8^+^ T-cells, which is paralleled by regain of TCR density on CD8^+^ lymphocytes and, apparently, restored the capacity to respond to new antigenic stimuli. Indeed, PTX therapy increased the number of CD8^+^IFNγ^+^ responsive to ASP2 *T*. *cruzi* antigen, while disfavored Pfn^+^ cells inside the heart tissue. Thus, PTX reestablished several aspects of the CD8^+^ T-cell alterations in chronically *T*. *cruzi*-infected mice. Considering that immunological abnormalities may contribute to cardiac alterations in experimental CCC [15, 54], we expected that the PTX-induced amelioration of the immunological unbalance in chronic *T*. *cruzi* infection would beneficially reverberate in the cardiac injury and dysfunction.

A loss of Cx43, the most abundant ventricular gap junction protein, is associated with arrhythmogenic disease [55]. The Cx43 loss may contribute to electrical conduction abnormalities in Chagas’ heart disease [56]. One important beneficial effect of PTX was the restoration of gap-junction Cx43 expression in chronically infected mice, therefore, indicating that Cx43 loss may be interrupted. In CD, overdeposition of FN discloses cardiac fibrosis [57]. PTX therapy hampered the progression of FN overexpression in experimental CCC; reinforcing the idea that in *T*. *cruzi* infection cardiac fibrosis can be improved, and even, reversed [15, 54, 58, 59]. The increased CK-MB activity in the serum, an important CCC feature and a biomarker of cardiomyocyte lesions [60], is increased in chronically infected mice before therapy, at 120 dpi [9]. PTX therapy also reduced and, moreover, reversed the increased CK-MB activity in the serum of experimental CCC. These findings support a broad beneficial effect of PTX on major features of *T*. *cruzi*-triggered heart injury.

To our knowledge, this study is the first demonstration that PTX improves electrical conduction and heart dysfunction in an infectious cardiomyopathy. In chronically infected C57BL/6 mice showing signs of CCC [9], PTX therapy ameliorated bradycardia, prolonged P wave duration, PR and QRS intervals, ART and AVB2. As previous shown in patients [2, 61] and *T*. *cruzi*-infected rhesus monkeys [59], higher ECG QRS scores directly correlated with the severity of heart fibrosis. Considering that electrical abnormalities and FN overdeposition were detected at 120 dpi [9,54], when therapy was initiated, our data support that PTX more than hampering progression is reversing electrical abnormalities and heart injury in experimental CCC. Moreover, the beneficial effects of PTX therapy were not restricted to a particularly experimental model, as amelioration of heart injury and electrical alterations were also observed in Colombian-infected C3H/He mice, a model of severe CCC [22]. Lastly, ECO studies revealed anatomical alterations with increased RV and LV areas, higher LV mass and decreased LVEF in chronically Colombian-infected mice, when compared with age-matched NI controls. Dilatation of the RV is a risk factor for sudden death in several cardiac diseases [62]. The enlargement of the RV, a marker for CCC in mice [63,64], was reduced by PTX therapy in chronic experimental CD. Increased LV internal dimensions emerged as a risk factor associated with morbidity and mortality in CD [65]. Further, increased LV mass, a marker of hypertrophy, is an independent risk factor of cardiovascular events [66]. Remarkably, PTX administration to mice with signs of CCC ameliorated the alterations in LV geometry and mass. Moreover, the reduced LVEF seen in chronically infected mice was improved to values detected in age-matched NI controls after PTX therapy. Similarly, PTX therapy improved LVEF in patients with heart failure due to ischemic cardiomyopathy [18,19]. Altogether, these data support that PTX therapy in chronic experimental CCC bettered cardiac tissue injury, electrical abnormalities and heart failure.

Here we bring evidence that immunological unbalance and Chagas’ heart disease are interconnected, involving multifactorial elements that may be working bidirectionally [9,15,22,33]. Further, whether the beneficial effects of PTX results of its immunomodulatory properties or direct action on heart tissue, particularly on cardiomyocytes, remains to be clarified. Therefore, our results opened a new avenue to be paved to explore PTX as adjuvant to immune protective response or cardioprotective tool in an infectious cardiomyopathy. More important, PTX emerges as a potent adjuvant to treat heart failure in CD. PTX might be a non fantasious strategy for CD immunotherapy, combined or not with trypanocidal drug, hampering the deleterious inflammation but preserving the beneficial anti-parasite immunity.

## Supporting Information

S1 Fig *Trypanosoma cruzi*-induced splenomegaly was partially reversed by PTX therapy. (A) Experimental design of the infection of C57BL/6 mice with 100 bt of the Colombian *T*. *cruzi* strain, treated daily with PTX from 120 to 150 dpi. (B) Kaplan-Meier curve represents the percentages of surviving mice. (C) Relative spleen weight (mg of spleen/g of body). *** *p<*0.001, saline-injected *T*. *cruzi*-infected mice compared with noninfected (NI) controls. The results represent ten to thirteen mice per experimental group in three independent experiments. ^#^ *p<*0.05, saline-injected compared with PTX-treated *T*. *cruzi*-infected mice.
(TIF)

S2 Fig PTX did not alter TNF production but reduced TNFR1/2 expression in *Trypanosoma cruzi* infection. (A) CBA for detection of TNF concentration in the serum. (B) qRT-PCR for detection of TNF mRNA in the heart tissue. (C) Frequency of CD8^+^TNF^+^ T-cell subsets in spleen. (D) Frequency of CD8^+^CD120a^+^ (TNFR1) and CD8^+^CD120b^+^ (TNFR2) T-cell subsets in spleen. The results represent three to five mice per experimental group in three independent experiments.* *p<*0.05, ** *p<*0.01 and *** *p<*0.001, saline-injected *T*. *cruzi*-infected mice compared with noninfected (NI) controls. ^#^ *p<*0.05 and ^###^ *p<*0.001, saline-injected compared with PTX-treated *T*. *cruzi*-infected mice.
(TIF)

S3 Fig PTX therapy reduced the frequency of cytotoxic CD8^+^ T-cells in chronically infected mice. Representative dot-plots of splenic CD8^+^ T-cells expressing IFNγ and CD107a in noninfected and saline-injected or PTX-treated *T*. *cruzi*-infected mice. The results show three mice per experimental group. The results represent three to five mice per experimental group.
(TIF)

S4 Fig PTX effects on trypomastigote forms of *T*. *cruzi* and heart parasitism. (A) Number of viable trypomastigote forms after 24 hours of *in vitro* treatment with different concentrations of PTX or the trypanocidal drug benznidazole (Bz; 10 μM) used as positive control. Data were obtained from three independent experiments. (B) Group data for immunohistochemistry detection of parasite nests in the heart tissue. (C) qPCR detection of *T*. *cruzi* Sat-DNA in the heart tissue of Colombian-infected C57BL/6 mice. The results represent three to five mice per experimental group. *** *p<*0.001, Bz- treated compared with not-treated (NT) trypomastigote forms.
(TIF)

S5 Fig PTX therapy has beneficial effects in a model of severe chronic Chagas’ heart disease. (A) Quantification of the Cx43-containing gap junction distances and FN-stained area (%) detected by IHC in the heart tissue, at 150 dpi. (B) Evaluation of CK-MB activity in serum, at 150 dpi. (C) Group data for ECG records showing P duration and PR, QTc and QRS intervals, at 150 dpi. (D) Summary of the group data from non-infected (NI) and infected mice showing the proportions of mice afflicted by arrhythmias (ART), second degree atrio-ventricular block (AVB2) and any ECG alterations, at 150 dpi. Representative data from two independent experiments. * *p<*0.05, ** *p<*0.01 and *** *p<*0.001, saline-injected *T*. *cruzi*-infected mice compared with NI controls. ^#^ *p<*0.05, ^##^ *p<*0.01 and ^###^ *p<*0.001, saline-injected compared with PTX-treated *T*. *cruzi*-infected mice.
(TIF)
