# Supplementary figures and images for: Pentoxifylline Reverses Chronic Experimental Chagasic Cardiomyopathy in Association with Repositioning of Abnormal CD8+ T-Cell Response
Source: PLoS Negl Trop Dis. 2015 Mar 19;9(3):e0003659. doi: 10.1371/journal.pntd.0003659 (PMC4366205; doi:10.1371/journal.pntd.0003659)

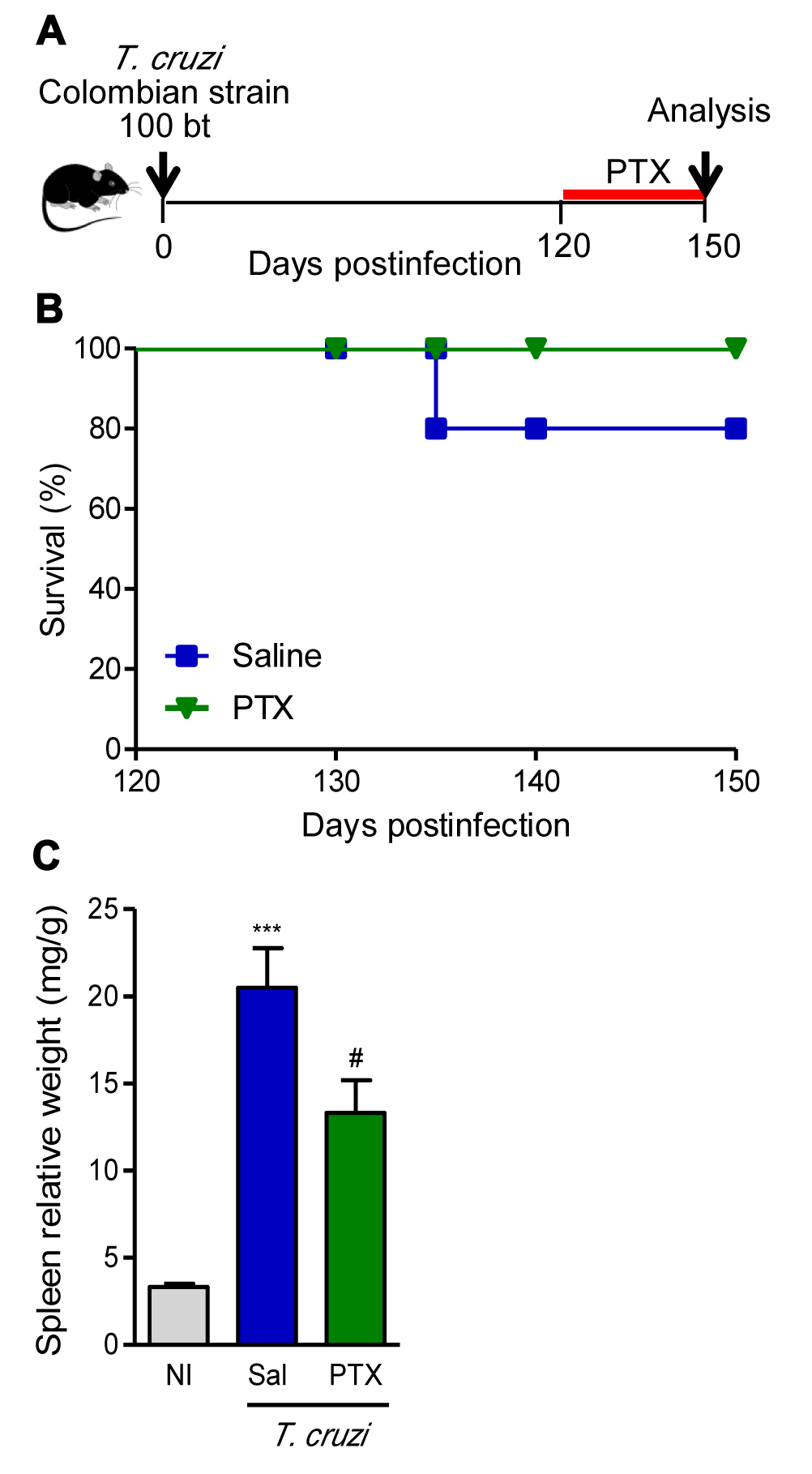

Supplement: S1 Fig — (A) Experimental design of the infection of C57BL/6 mice with 100 bt of the Colombian T. cruzi strain, treated daily with PTX from 120 to 150 dpi. (B) Kaplan-Meier curve represents the percentages of surviving mice. (C) Relative spleen weight (mg of spleen/g of body). *** p<0.001, saline-injected T. cruzi-infected mice compared with noninfected (NI) controls. The results represent ten to thirteen mice per experimental group in three independent experiments. # p<0.05, saline-injected compared with PTX-treated T. cruzi-infected mice. (TIF) [file pntd.0003659.s001.tif]

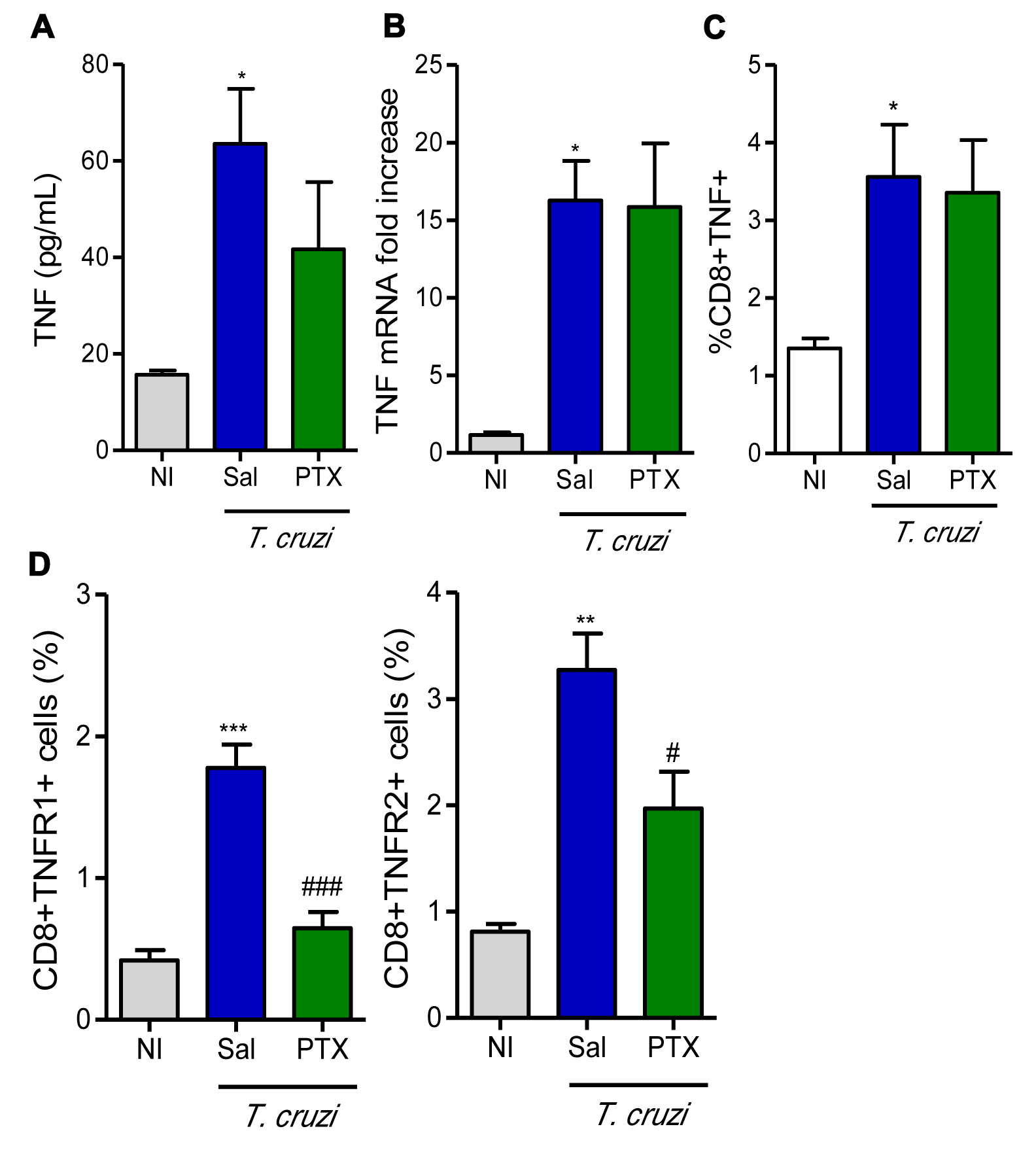

Supplement: S2 Fig — (A) CBA for detection of TNF concentration in the serum. (B) qRT-PCR for detection of TNF mRNA in the heart tissue. (C) Frequency of CD8+TNF+ T-cell subsets in spleen. (D) Frequency of CD8+CD120a+ (TNFR1) and CD8+CD120b+ (TNFR2) T-cell subsets in spleen. The results represent three to five mice per experimental group in three independent experiments.* p<0.05, ** p<0.01 and *** p<0.001, saline-injected T. cruzi-infected mice compared with noninfected (NI) controls. # p<0.05 and ### p<0.001, saline-injected compared with PTX-treated T. cruzi-infected mice. (TIF) [file pntd.0003659.s002.tif]

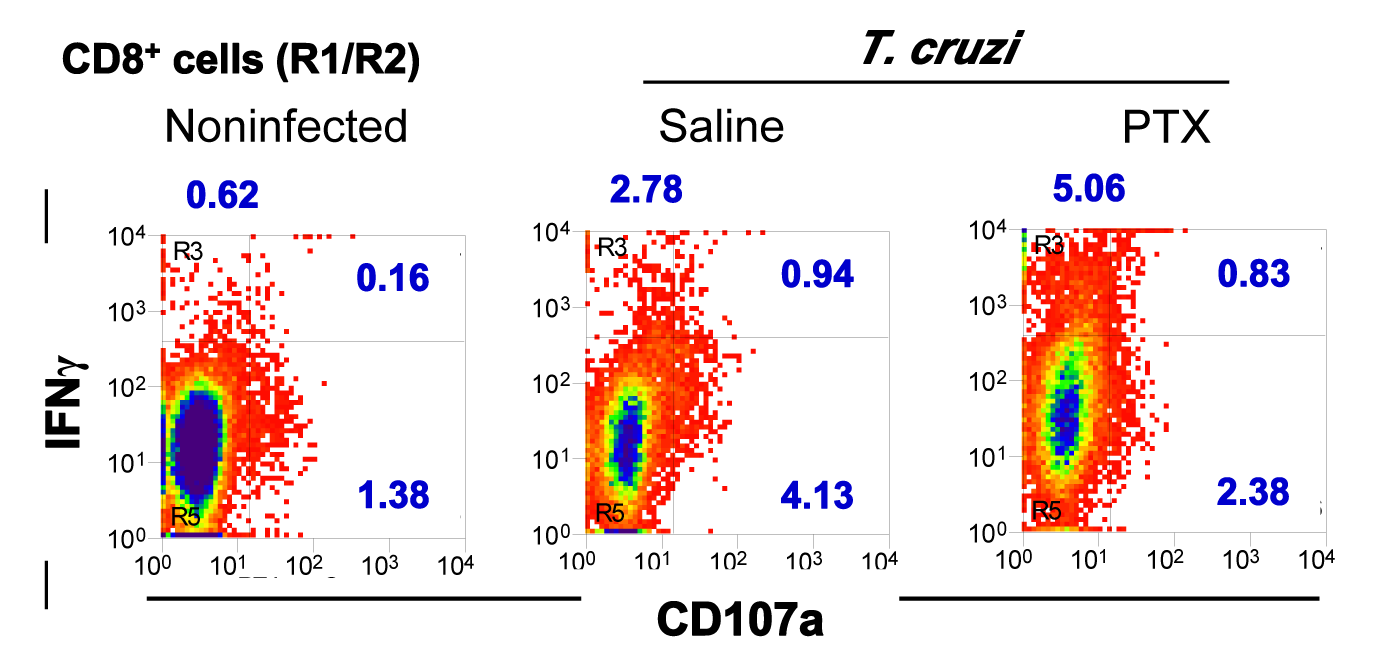

Supplement: S3 Fig — Representative dot-plots of splenic CD8+ T-cells expressing IFNγ and CD107a in noninfected and saline-injected or PTX-treated T. cruzi-infected mice. The results show three mice per experimental group. The results represent three to five mice per experimental group. (TIF) [file pntd.0003659.s003.tif]

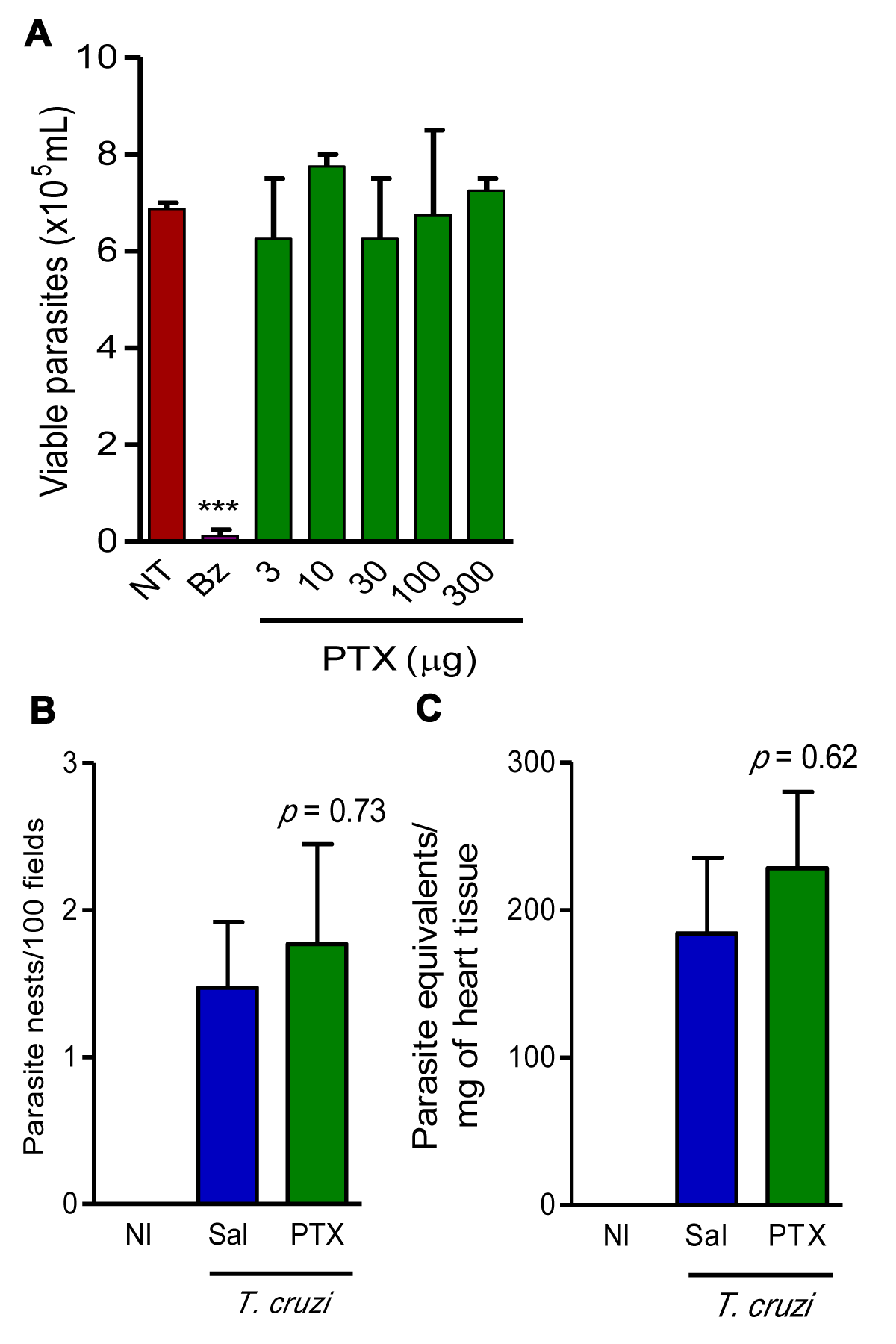

Supplement: S4 Fig — (A) Number of viable trypomastigote forms after 24 hours of in vitro treatment with different concentrations of PTX or the trypanocidal drug benznidazole (Bz; 10 μM) used as positive control. Data were obtained from three independent experiments. (B) Group data for immunohistochemistry detection of parasite nests in the heart tissue. (C) qPCR detection of T. cruzi Sat-DNA in the heart tissue of Colombian-infected C57BL/6 mice. The results represent three to five mice per experimental group. *** p<0.001, Bz- treated compared with not-treated (NT) trypomastigote forms. (TIF) [file pntd.0003659.s004.tif]

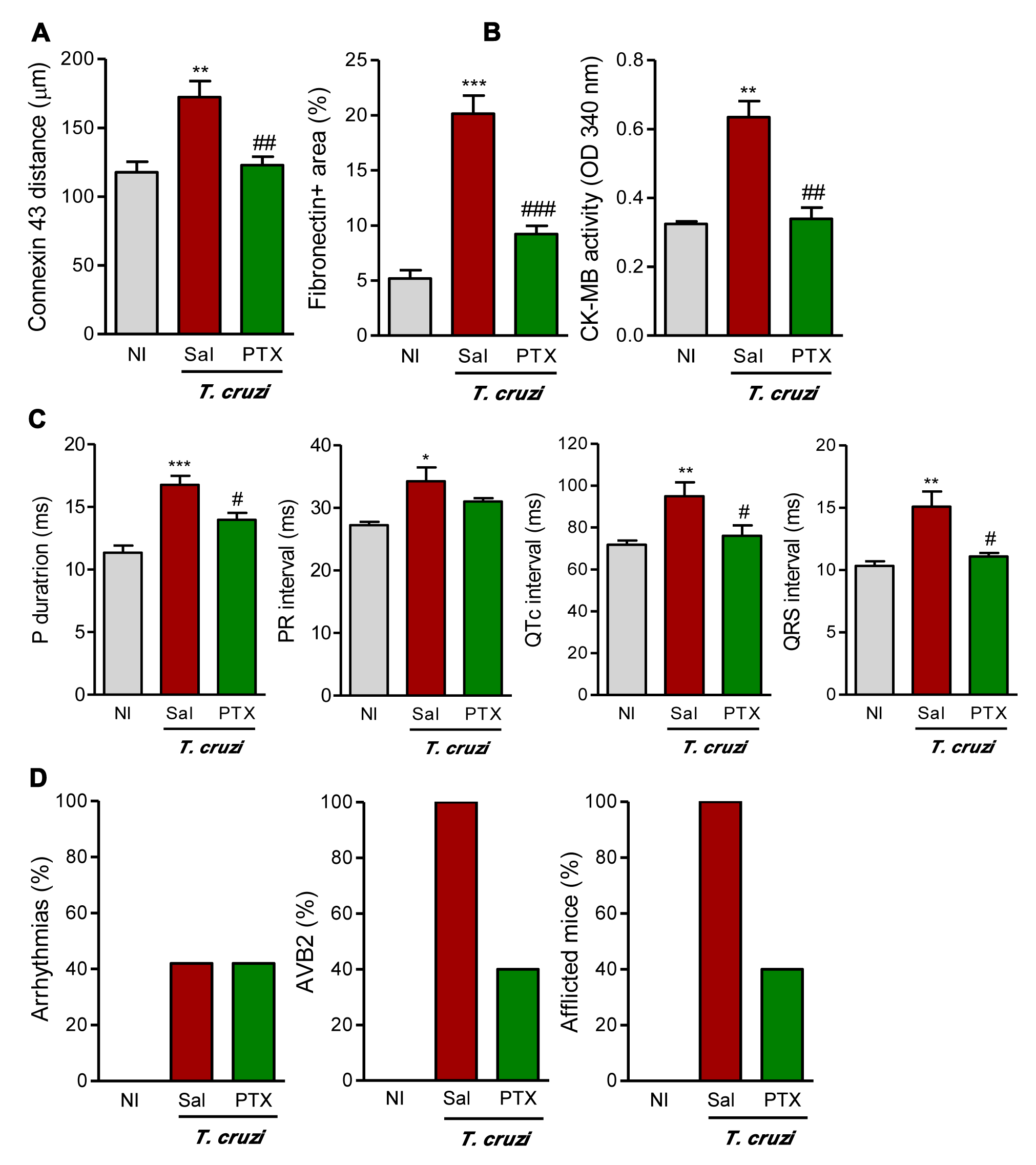

Supplement: S5 Fig — (A) Quantification of the Cx43-containing gap junction distances and FN-stained area (%) detected by IHC in the heart tissue, at 150 dpi. (B) Evaluation of CK-MB activity in serum, at 150 dpi. (C) Group data for ECG records showing P duration and PR, QTc and QRS intervals, at 150 dpi. (D) Summary of the group data from non-infected (NI) and infected mice showing the proportions of mice afflicted by arrhythmias (ART), second degree atrio-ventricular block (AVB2) and any ECG alterations, at 150 dpi. Representative data from two independent experiments. * p<0.05, ** p<0.01 and *** p<0.001, saline-injected T. cruzi-infected mice compared with NI controls. # p<0.05, ## p<0.01 and ### p<0.001, saline-injected compared with PTX-treated T. cruzi-infected mice. (TIF) [file pntd.0003659.s005.tif]
